# High-sensitive C-reactive protein and risk of incident type 2 diabetes: a case–control study nested within the Singapore Chinese Health Study

**DOI:** 10.1186/s12902-017-0159-5

**Published:** 2017-02-08

**Authors:** An Pan, Yeli Wang, Jian-Min Yuan, Woon-Puay Koh

**Affiliations:** 1 Department of Epidemiology and Biostatistics, Ministry of Education Key Lab of Environment and Health, School of Public Health, Tongji Medical College, Huazhong University of Science and Technology, Wuhan, 430030 Hubei China; 2 Saw Swee Hock School of Public Health, National University of Singapore and National University Health System, Singapore, Singapore; 3 Division of Cancer Control and Population Sciences, University of Pittsburgh Cancer Institute, Pittsburgh, PA USA; 4 Department of Epidemiology, University of Pittsburgh Graduate School of Public Health, Pittsburgh, PA USA; 5 Duke-NUS Medical School Singapore, Singapore, Singapore

**Keywords:** Type 2 diabetes, C-reactive protein, Inflammation, Prospective studies

## Abstract

**Background:**

The liver-derived C-reactive protein (CRP) is a sensitive and systemic biomarker of inflammation, and has been associated with increased risk of developing type 2 diabetes in populations other than Chinese. Therefore, we prospectively examined the relation between plasma levels of CRP and risk of type 2 diabetes (T2D) among a Chinese population.

**Methods:**

Plasma high-sensitive CRP (hs-CRP) concentrations were assayed among 571 T2D cases and 571 controls nested in the prospective cohort of the Singapore Chinese Health Study. Both cases and controls were free of physician-diagnosed diabetes, cardiovascular disease and cancer at blood collections (1999–2004). Incident physician-diagnosed T2D cases were self-reported during the follow-up visits (2006–2010), and controls were matched for age (±3 years) and date (±6 months) of blood collection and gender. Multivariable logistic regression models were used to compute the odds ratio (OR) and the corresponding 95% confidence intervals (CIs).

**Results:**

The mean (SD) concentrations of hs-CRP were 2.79 (2.65) and 1.86 (2.03) mg/L, respectively, in cases and controls (*P* < 0.001). After multivariate adjustment for T2D risk factors such as lifestyle, body mass index, plasma triglycerides and HDL cholesterol, the OR comparing the extreme quartiles of hs-CRP was 1.74 [95% CI 1.12–2.70; *P* for trend = 0.016]. When the analysis was limited to 279 cases who had HbA1c ≥6.5% at the time of blood collection and their controls, the OR comparing the extreme quartiles of hs-CRP was 2.43 (95% CI 1.25–4.71; *P* for trend = 0.003). When confined to the other 292 subjects with HbA1c <6.5% and their controls, the corresponding OR was 1.24 (95% CI 0.64–2.39; *P* for trend = 0.93).

**Conclusions:**

We found that CRP was not associated with increased risk of incident diabetes in this cohort of Chinese in Singapore. Previous positive findings from prospective studies might be partly due to undiagnosed T2D among the cases during blood collection.

**Electronic supplementary material:**

The online version of this article (doi:10.1186/s12902-017-0159-5) contains supplementary material, which is available to authorized users.

## Background

C-reactive protein (CRP) is synthesized by the liver and has been shown to be a sensitive and systemic biomarker of inflammation [1]. A number of prospective cohort studies and nested case–control studies have reported that CRP is associated with increased risk of developing type 2 diabetes (T2D) [2]. A recent meta-analysis of 18 prospective studies found that the overall relative risk (RR) of T2D was 1.26 (95% confidence interval [CI] 1.16–1.37) per 1 log mg/L increment in CRP levels [2]. However, most of the studies have been conducted in US or European populations [2], and two studies were done in Japanese adults [3, 4]. To the best of our knowledge, no study has specifically investigated the relation between CRP and risk of incident T2D in a Chinese population.

A few cross-sectional studies have been conducted in Chinese population, and suggest that CRP is positively related to prediabetes (including hyperglycemia and metabolic syndrome) and prevalent diabetes [5–11]. However, the temporal relations cannot be determined in cross-sectional studies and reverse causality is a major concern. The positive relation in cross-sectional studies or case–control studies could be due to CRP being a consequence of hyperglycemia. Therefore, a prospective study is needed to ascertain the elevation of CRP before the onset of hyperglycaemia in the development of T2D. Ye et al. [12] recently included elevated CRP levels in a prediction model of incident T2D in a 6-year follow-up study of 1912 Chinese adults aged 50–70 years, but the exact association between CRP and diabetes risk was not reported. Studies have consistently shown that circulating CRP levels are generally lower among Asians than Caucasians and Hispanic populations [5, 13, 14]. Therefore, it is of scientific interest whether CRP could predict the onset of diabetes in Chinese population with relatively lower levels. In this prospective, nested, case–control study, we examined the role of CRP in predicting the development of incident T2D independent of obesity, lifestyle and blood lipid profiles in Chinese adults.

## Methods

### Study population

The design of the Singapore Chinese Health Study (SCHS) has been described previously [15]. Briefly, the SCHS was established between 1993 and 1998 when 63,257 Chinese adults aged 45–74 years residing in Singapore responded to an in-person interview including questions on usual diet, demographics, height and weight, smoking and drinking habits, usual physical activity, and medical history including physician diagnosed diabetes, hypertension, coronary heart disease, stroke and cancer. A total of 52,322 participants were successfully re-contacted via telephone between 1999 and 2004 (follow-up I) to update certain lifestyle practices (e.g., smoking habits and alcohol consumption), body weight and medical history (e.g., diabetes, hypertension, coronary heart disease, and stroke). They were also invited to donate their blood samples and 32,535 participants agreed and gave their morning blood samples. The current study used the date of blood sample collection at follow-up I visits as the baseline. Participants (*n* = 39,528) were re-contacted again in 2006–2010 (follow-up II) to update their lifestyle habits and medical history, and among them, 25,477 donated blood samples at follow-up I. The study has been approved by the Institutional Review Boards at the National University of Singapore and University of Pittsburgh, and informed consent was provided with completion of the baseline interview.

### Ascertainment of diabetes

History of physician-diagnosed diabetes was asked in baseline questionnaires administered by a trained interviewer. The diabetes status was inquired again by the following question asked during the first and second follow-up telephone interviews: “Have you been told by a doctor that you have diabetes (high blood sugar)?” If the answer was “yes”, participants were also asked for the age at which they were first diagnosed. The prevalent diabetes cases were those who reported to have diabetes at baseline or follow-up I visits, and the incident diabetes were those who reported to have diabetes only at follow-up II visits and after the donation of blood specimens. The robustness and accuracy of the self-reported diabetes cases was validated in another study analyzing 1651 cohort participants using two complementary methods [16]: 949 diabetes cases were validated by a hospital-based discharge summary database and 702 cases via a supplementary questionnaire regarding symptoms, diagnostic tests and diabetes treatment during the telephone interview. A positive predictive value of 99% was found in the validation study, suggesting that the self-reported history of diabetes was a reliable measure of diabetes status of the study population.

### Assessment of confounders

Potential confounders were evaluated at baseline and follow-up I visits. The body mass index (BMI) was calculated as self-reported body weight in kilograms divided by the square of height in meters. The self-reported BMI has been found to be linearly associated with risk of diabetes in this cohort [17]. Smoking status was coded as never, former and current smoking, and alcohol consumption was coded as never/occasional, weekly and daily drinking based on the follow-up I questionnaires. Education level (no, primary school, secondary school and above) and moderate physical activity levels (<0.5, 0.5–3.9, and ≥4.0 hours/week) were obtained from baseline questionnaires.

### Selection of cases and controls

For the current analysis, we established a nested case–control study of 571 cases and 571 matched controls within SCHS. All cases and controls were free of physician-diagnosed diabetes, cardiovascular disease and cancer at baseline interview as well as at blood collection (1999–2004). Incident self-reported T2D cases were identified during the follow-up II visits during 2006–2010, and controls who were free of diabetes and cardiovascular disease were matched, at the 1:1 ratio, with the cases on age (±3 years) and date (±6 months) of blood collection, sex and dialect group. In addition, all selected controls were tested for hemoglobin A1c (HbA1c) to identify undiagnosed T2D. All subjects HbA1c ≥6.0% were ineligible for the study and a replacement control with the same matching criteria was randomly chosen among the remaining eligible subjects. The study flow is shown in Additional file 1: Figure S1.

### Laboratory procedures

A 20-mL peripheral blood sample was obtained from each consenting subject. Immediately after blood collection, the tubes were put on ice during transport from the subjects’ homes to the laboratory. All of the specimens were then separated into various components (plasma, serum, red blood cells, and buffy coat). All of the specimens were subsequently stored in −80 °C freezers for long-term storage. Frozen plasma aliquots from case and control subjects were selected for simultaneous analysis at the same batch at the National University Hospital Reference Laboratory. Plasma hs-CRP levels were measured via colorimetric method on a chemistry analyzer (AU5800 Analyzer, Beckman Coulter, Brea, CA). The within-assay and between-assay coefficients of variation (CV) were 0.5–1.4% and 1.0–1.6%, respectively. Blood lipids [total cholesterol, triglycerides (TG) and HDL cholesterol (HDL-C)] were also measured via colorimetric method on the AU 5800 system, and the within-assay and between-assay CVs were all less than 1.3%. Hemoglobin A1c (HbA1c) was measured by HPLC method using Bio-Rad Variant II™ System (Bio-Rad Laboratories, Hercules, CA) in red blood cells.

### Statistical analysis

Study participants were divided into quartiles according to the distribution of hs-CRP levels among control subjects, and the lowest quartile served as the reference group. We used conditional logistic regression to model the CRP-T2D association with adjustment for age (continuous), BMI (continuous), smoking status, physical activity, alcohol use, history of hypertension, plasma TG and HDL-C levels. Tests of linear trend across increasing quartiles were conducted using the median value of each quartile and treating it as a continuous variable. We also calculated the risk of T2D associated with per 1 log mg/L increment in hs-CRP levels, in order to compare our results with previous ones [2]. Some previous studies have suggested potential sex differences in the association [18], thus, we tested the interaction by including the interaction term of log hs-CRP levels (continuous) and sex in the model, and then conducted a stratified analysis by sex using sex-specific quartiles. Similar stratified analysis was also done by baseline BMI status (<23 and ≥23 kg/m^2^), and in this stratified analysis, unconditional logistic regression models were used with further adjustment for sex and dialect. All P values were two-sided. Data were analyzed with STATA version 14 (Stata Corp, College Station, Texas).

## Results

Among T2D cases, the mean (±SD) age at diagnosis was 63.2 ± 6.4 years and the mean (±SD) duration between blood donation and diagnosis of T2D was 4.0 ± 1.7 years. Characteristics of study participants assessed at blood collections (1999–2004) are shown in Table 1. The mean age of the participants was 59.7 (SD 6.2) years, and 41.3% were males. As expected, diabetic participants had high-risk profiles except for the matching factors. They were heavier, were more likely to have history of hypertension than control subjects. No significant differences were found for education level, smoking status, alcohol drinking and physical activity levels. Regarding the plasma biomarkers, diabetic cases had higher levels of HbA1c, random glucose and insulin, and triglycerides, but lower HDL-cholesterol levels. The mean (SD) concentration of hs-CRP was 2.79 (2.65) and 1.86 (2.03) mg/L, respectively, in cases and controls (*P* < 0.001). Among the healthy control participants, hs-CRP was inversely correlated with levels of HDL-C (Pearson’s coefficient *r* = −0.15), and positively correlated with TG levels and BMI (Pearson’s coefficient *r* = 0.11 and 0.26, respectively) (data not shown).

**Table 1:** Characteristics of the diabetes cases and matched controls: The Singapore Chinese Health Study

Characteristics	Cases	Controls	*p* value^*^
Number of participants	571	571	-
Age, years	59.6 ± 6.1	59.7 ± 6.2	0.78
Gender (Male)	236 (41.3)	236 (41.3)	-
Dialect (%)			-
Cantonese	287 (50.3)	287 (50.3)	
Hokkien	284 (49.7)	284 (49.7)	
Body mass index, kg/m^2^	24.8 ± 3.6	22.8 ± 3.3	<0.001
Level of education (%)			0.15
No formal education	104 (18.2)	99 (17.3)	
Primary school	255 (44.7)	233 (40.8)	
Secondary school or higher	212 (37.1)	239 (41.9)	
Cigarette smoking (%)			0.08
Never smokers	410 (71.8)	425 (74.4)	
Ever smokers	161 (28.2)	146 (25.6)	
History of hypertension (%)	265 (46.1)	148 (25.9)	<0.001
Weekly moderate-to-vigorous activity (%)			0.11
< 0.5 hours/week	456 (79.9)	454 (79.5)	
0.5–3.9 hours/week	82 (14.4)	68 (11.9)	
≥ 4 hours/week	33 (5.8)	49 (8.6)	
Alcohol intake (%)			0.81
Abstainers	498 (87.2)	497 (87.0)	
Weekly drinkers	55 (9.6)	59 (10.3)	
Daily drinkers	18 (3.2)	15 (2.6)	
C-reactive protein, mg/L	2.79 ± 2.65	1.86 ± 2.03	<0.001
HbA1c, %	6.83 ± 1.44	5.55 ± 0.27	<0.001
Random glucose, mmol/L	7.27 ± 3.65	4.84 ± 1.22	<0.001
Random insulin, mIU/L	27.8 ± 33.8	18.0 ± 22.5	<0.001
Total cholesterol, mmol/L	5.31 ± 0.95	5.20 ± 0.85	0.049
HDL cholesterol, mmol/L	1.08 ± 0.24	1.23 ± 0.32	<0.001
Triglyceride, mmol/L	2.44 ± 1.53	1.80 ± 1.04	<0.001

Data are expressed as mean ± standard deviation for continuous variables and n (percentage) for categorical variables

Cases and controls are matched on age at blood taken (±3 years), gender, dialect, and date of blood collection (±6 months)

^*^ *p* values based on the chi-square test for categorical variables and Student’s *t*-test for continuous variables

**Table 2:** Risk of diabetes according to quartiles of hs-CRP: The Singapore Chinese Health Study

	Quartiles of hs-CRP^a^				*P* for trend^b^	Per 1 log mg/L increase
	Q1	Q2	Q3	Q4		
Median (range)	0.4 (0.1–0.6)	0.9 (0.7–1.2)	1.6 (1.3–2.3)	3.6 (2.4–11.0)		
Total diabetes						
Cases/controls	78/154	107/139	150/138	236/140		
Model 1	1.00	1.51 (1.03–2.20)	2.14 (1.47–3.11)	3.25 (2.28–4.64)	<0.001	1.59 (1.40–1.81)
Model 2	1.00	1.42 (0.96–2.11)	1.89 (1.27–2.80)	2.70 (1.85–3.95)	<0.001	1.50 (1.31–1.71)
Model 3	1.00	1.23 (0.81–1.86)	1.50 (0.99–2.27)	1.97 (1.32–2.94)	<0.001	1.34 (1.16–1.54)
Model 4	1.00	1.24 (0.79–1.96)	1.22 (0.77–1.92)	1.74 (1.12–2.70)	0.016	1.27 (1.09–1.48)
Undiagnosed diabetes						
Cases/controls	32/81	43/67	73/54	131/77		
Model 1	1.00	1.48 (0.84–2.62)	3.63 (2.02–6.53)	4.39 (2.59–7.45)	<0.001	1.84 (1.52–2.24)
Model 2	1.00	1.29 (0.71–2.37)	3.17 (1.72–5.82)	3.63 (2.08–6.36)	<0.001	1.75 (1.43–2.14)
Model 3	1.00	1.21 (0.64–2.28)	2.41 (1.26–4.59)	2.55 (1.42–4.58)	<0.001	1.53 (1.24–1.89)
Model 4	1.00	1.20 (0.59–2.44)	2.17 (1.07–4.40)	2.43 (1.25–4.71)	0.003	1.53 (1.20–1.94)
Incident diabetes						
Cases/controls	46/73	64/72	77/84	105/63		
Model 1	1.00	1.45 (0.87–2.43)	1.45 (0.88–2.39)	2.54 (1.54–4.20)	<0.001	1.38 (1.16–1.64)
Model 2	1.00	1.50 (0.85–2.65)	1.24 (0.70–2.18)	2.20 (1.26–3.84)	0.008	1.31 (1.08–1.59)
Model 3	1.00	1.31 (0.73–2.36)	1.07 (0.60–1.93)	1.70 (0.95–3.07)	0.14	1.20 (0.98–1.47)
Model 4	1.00	1.24 (0.65–2.39)	0.75 (0.39–1.45)	1.24 (0.64–2.39)	0.93	1.06 (0.85–1.33)

*Abbreviation: hs-CRP* high-sensitive C-reactive protein, *OR* odds ratio, *CI* confidence interval

^a^Quartiles of hs-CRP were created based on controls in the whole population

^b^Linear trend was tested by using the median level of each quartile of hs-CRP as continuous variables

Model 1: adjusted for age (continuous variable), matched on age (±3 years), sex, dialect, and date of blood collection (±6 months)

Model 2: model 1 plus education level, smoking status, alcohol intake, physical activity, hypertension, and fasting status

Model 3: model 2 plus adjusted for body mass index (continuous)

Model 4: model 3 plus plasma triglycerides and HDL-cholesterol levels (both in quartiles)

After multivariate adjustment for demographic and lifestyle factors, the odds ratio (OR) comparing the extreme quartiles of hs-CRP was 2.70 (95% CI 1.85–3.95; *P* for trend <0.001; Table 2). Further adjustment for BMI, plasma levels of TG and HDL-C attenuated the association but it remained significant (OR = 1.74 comparing the extreme quartiles of hs-CRP; 95% CI 1.12–2.70; *P* for trend = 0.016). Among the cases, 279 subjects had HbA1c ≥6.5% at the time of blood collection and the OR comparing the extreme quartiles of hs-CRP was 2.43 (95% CI 1.25–4.71; *P* for trend = 0.003). The other 292 subjects had HbA1c <6.5% at blood collection and the corresponding OR was 1.24 (95% CI 0.64–2.39; *P* for trend = 0.93).

The OR (95% CI) for T2D of each 1 log mg/L increment in hs-CRP levels was 1.27 (1.09–1.48) in the total study samples, 1.53 (1.20–1.94) in those with HbA1c ≥6.5% at blood collection, and 1.06 (0.85–1.33) in those with HbA1c <6.5% at blood collection (Table 2).

We further stratified the analysis by sex (Table 3) and baseline BMI status (Table 4). The association was slightly stronger in women compared to men, but the interaction was not statistically significant (*P* for interaction = 0.27). The association was similar in normal weight individuals (BMI <23 kg/m^2^) and overweight/obese participants (BMI ≥23 kg/m^2^), and the interaction test was not significant (*P* for interaction = 0.72).

**Table 3:** Risk of diabetes according to sex-specific quartiles of hs-CRP: stratified by sex

	Quartiles of hs-CRP^a^				*P* for trend^b^	Per 1 log mg/L increase
	Q1	Q2	Q3	Q4		
Men						
Median (range)	0.3 (0.12–0.4)	0.8 (0.5–1.1)	1.5 (1.2–2.0)	3.4 (2.1–11.0)		
Cases/controls	22/60	70/64	59/53	85/59		
Model 1	1.00	2.79 (1.54–5.05)	3.00 (1.60–5.63)	3.73 (2.04–6.79)	0.002	1.46 (1.21–1.75)
Model 2	1.00	3.42 (1.77–6.61)	3.06 (1.53–6.13)	3.57 (1.80–7.11)	0.039	1.38 (1.12–1.70)
Model 3	1.00	2.76 (1.40–5.47)	2.45 (1.20–5.03)	2.80 (1.38–5.69)	0.029	1.30 (1.05–1.61)
Model 4	1.00	2.86 (1.36–6.01)	1.90 (0.86–4.19)	2.25 (1.06–4.79)	0.24	1.19 (0.94–1.49)
Women						
Median (range)	0.5 (0.12–0.7)	1.1 (0.8–1.3)	1.8 (1.4–2.5)	4.3 (2.6–11.0)		
Cases/controls	44/94	56/79	87/82	148/80		
Model 1	1.00	1.53 (0.91–2.57)	2.29 (1.38–3.80)	3.86 (2.39–6.21)	<0.001	1.72 (1.44–2.04)
Model 2	1.00	1.37 (0.79–2.37)	2.00 (1.16–3.46)	3.32 (1.99–5.53)	<0.001	1.63 (1.36–1.96)
Model 3	1.00	1.10 (0.61–2.00)	1.45 (0.80–2.66)	2.19 (1.24–3.85)	0.002	1.42 (1.16–1.73)
Model 4	1.00	1.09 (0.54–2.20)	1.25 (0.63–2.47)	2.07 (1.07–3.99)	0.012	1.41 (1.12–1.78)

*Abbreviation: hs-CRP* high-sensitive C-reactive protein, *OR* odds ratio, *CI* confidence interval

^a^Quartiles of hs-CRP were created separately for men and women among controls

^b^Linear trend was tested by using the median level of each quartile of hs-CRP as continuous variables

Model 1: adjusted for age (continuous variable), matched on age (±3 years), sex, dialect, and date of blood collection (±6 months)

Model 2: model 1 plus education level, smoking status, alcohol intake, physical activity, hypertension, and fasting status

Model 3: model 2 plus adjusted for body mass index (continuous)

Model 4: model 3 plus plasma triglycerides and HDL-cholesterol levels (both in quartiles)

**Table 4:** Risk of diabetes according to quartiles of hs-CRP: stratified by baseline BMI

	Quartiles of hs-CRP^a^				*P* for trend^b^	Per 1 log mg/L increase
	Q1	Q2	Q3	Q4		
Median (range)	0.4 (0.1–0.6)	0.9 (0.7–1.2)	1.6 (1.3–2.3)	3.6 (2.4–11.0)		
BMI <23 kg/m^2^						
Cases/controls	37/109	42/84	48/68	59/57		
Model 1	1.00	1.49 (0.88–2.53)	2.08 (1.23–3.51)	2.95 (1.75–4.99)	<0.001	1.47 (1.22–1.77)
Model 2	1.00	1.41 (0.82–2.43)	1.93 (1.12–3.32)	2.67 (1.54–4.60)	<0.001	1.42 (1.17–1.72)
Model 3	1.00	1.27 (0.71–2.25)	1.38 (0.77–2.47)	2.07 (1.16–3.70)	0.02	1.29 (1.05–1.58)
BMI ≥23 kg/m^2^						
Cases/controls	41/45	65/55	102/70	177/83		
Model 1	1.00	1.39 (0.79–2.44)	1.74 (1.02–2.96)	2.62 (1.56–4.41)	<0.001	1.55 (1.29–1.86)
Model 2	1.00	1.30 (0.73–2.33)	1.43 (0.82–2.50)	2.19 (1.28–3.78)	0.002	1.46 (1.21–1.77)
Model 3	1.00	1.11 (0.60–2.05)	1.20 (0.67–2.15)	1.68 (0.95–2.97)	0.041	1.33 (1.09–1.63)

*Abbreviation: hs-CRP* high-sensitive C-reactive protein, *BMI* body mass index, *OR* odds ratio, *CI* confidence interval

^a^Quartiles of hs-CRP were created based on controls in the whole population

^b^Linear trend was tested by using the median level of each quartile of hs-CRP as continuous variables

Model 1: adjusted for age (continuous variable), sex, and dialect

Model 2: model 1 plus education level, smoking status, alcohol intake, physical activity, hypertension, and fasting status

Model 3: model 2 plus plasma triglycerides and HDL-cholesterol levels (both in quartiles)

## Discussion

In this prospective nested case–control study of Chinese men and women, elevated baseline plasma CRP levels were associated with an increased risk of T2D. However, when stratified by baseline HbA1c levels, we found that CRP was only positively associated with T2D among those already with high HbA1c levels (undiagnosed diabetes), but not in those with low HbA1c levels (incident diabetes). Therefore, elevated CRP levels might be by-products of hyperglycemia, rather than directly contributing to the development of incident T2D.

HbA1c was adapted as a diagnosis criterion of diabetes in 2010 by the American Diabetes Association [19]; therefore, at the time of blood collection and follow-up in our cohort, HbA1c level was not used in the diagnosis of diabetes in Singapore. In the total study samples, irrespective of HbA1c levels in the cases, we observed a strong positive association between CRP and T2D. The estimate (OR = 1.27 [95% CI 1.09–1.48] per 1 log mg/L increment in CRP levels) was consistent with the pooled relative risk reported from a recent meta-analysis [1.26 (95% CI 1.16–1.37); 18 studies] [2]. None of the previous studies included HbA1c in their diagnosis criteria, and two prior studies in Caucasian populations have observed positive CRP-T2D associations among subgroup subjects with HbA1c <5.8% [20] and HbA1c <6.0% [21], respectively, which were contrary to the findings of the current study. Both studies have also adjusted for HbA1c in the statistical models, and the positive association between CRP and incident diabetes did not change materially [20, 21]. We did not adjust for HbA1c levels in our model, because we had purposely excluded controls with baseline HbA1c ≥6.0% to reduce the possibility of undiagnosed diabetes among the controls. Therefore, the cases had much higher HbA1c levels compared with the controls at the time of blood collection (Table 1), and adjustment for the Hb1Ac levels would be problematic due to its marked difference between cases and controls. Since no other studies have specifically evaluated the effect of high HbA1c levels at baseline among the incident diabetes cases, it is unclear to what extent the positive association in previous prospective reports could be explained by the effect of undiagnosed diabetes. Some studies also found no significant associations between CRP and incident diabetes in Pima Indians [22], UK adults [18], Aboriginal Canadians [23], Germany men [24], Mexican men [25], and US adults [26]. Several studies have suggested that CRP-diabetes association could be largely explained by obesity [23, 24, 26, 27], insulin resistance [26, 27], deranged liver function and lower adiponectin levels [18].

The stronger association between CRP and glycemia in Chinese women compared to men in some studies [7, 8, 11, 28] may be explained by the greater accumulation of subcutaneous fat in women than in men [29]. Two prospective cohort studies in Mexican [25] and German [30] populations observed positive CRP-T2D associations in women but not in men, while two cohort studies in Japanese populations [3, 4] found no significant gender differences in the association. In our study, although no significant interaction was observed between sex and CRP (*P* = 0.27), the association with T2D risk was stronger in women compared to men when CRP was examined as a continuous variable, and this finding is generally consistent with previous prospective studies [18].

Our finding of a positive association between CRP and increased risk of undiagnosed diabetes but not incident diabetes suggests that CRP might not be a causal factor for diabetes, but is a marker of hyerglycaemia in the pathway. Although the meta-analysis revealed a statistically significant increased diabetes risk associated with CRP [2], the results are not entirely consistent and a number of studies did not report any significant association either in the whole population [18, 22, 23, 26] or in men [24, 25]. The current evidence remains controversial whether CRP is a causal risk factor or just a downstream intermediate for T2D [31]. Hence, the clinical potential of targeting CRP in the prevention of diabetes remains uncertain. A recent Mendelian randomization analysis in the Whitehall II Study found that CRP haplotypes were not associated with incident diabetes despite the association with baseline serum CRP [32]. Other Mendelian randomization studies also found no causal relation between CRP and metabolic syndrome [33], as well as coronary heart disease [34–36]. Therefore, the lack of concordance between the effect of CRP genotypes and CRP levels on T2D and coronary heart disease risks argues against a causal role of CRP in the etiologies of these two diseases.

The strength of the present study was its prospective design and hence the presumed lack of recall bias in exposure data (questionnaires, collected biospecimens) prior to T2D diagnosis. However, there are some limitations to the present study as well. First, we measured CRP only once at baseline and this may not represent the long-term lipid profile. However, this would lead to non-differential misclassification and may underestimate the association. In addition, the BMI was calculated from self-reported height and weight, and residual confounding is possible. Second, incident diabetes was obtained from self-reported information, thus undiagnosed diabetes may exist. However, we have measured HbA1c levels, which was updated as a diagnosis criterion of diabetes in 2010 by the American Diabetes Association [19], and further performed stratified analysis among subgroups with HbA1c <6.5% and ≥6.5%. Furthermore, we have used HbA1c as a selection criterion for controls to minimize bias due to undiagnosed diabetes; therefore, we could not include it in our model adjustment. Last but not least, the present study was conducted in a middle-aged and elderly population with a higher diabetes incidence, therefore, the findings may not be generalizable to younger people.

## Conclusion

In conclusion, we found that elevated plasma levels of hs-CRP were only positively associated with T2D among those already with high HbA1c levels, but not in those with low HbA1c levels in this Chinese population. Therefore, elevated CRP levels may be a consequence of hyperglycemia, instead of being an etiological biomarker in T2D development. Current evidence remains controversial whether CRP is a causal risk factor or just a downstream intermediate for T2D. Therefore, more carefully constructed prospective studies in different populations are warranted to validate this finding, and investigate the biochemical and genetic basis for the relationship between hs-CRP and T2D risk.

## Additional file

Additional file 1: Figure S1. Flow chart of the Singapore Chinese Health Study. (DOCX 25 kb)

## Abbreviations

BMI: Body mass index
CI: Confidence interval
hs-CRP: high-sensitivity C-reactive protein
OR: Odds ratio
T2D: Type 2 diabetes
