# Supplementary material for: High-sensitive C-reactive protein and risk of incident type 2 diabetes: a case–control study nested within the Singapore Chinese Health Study
Source: BMC Endocr Disord. 2017 Feb 8;17:8. doi: 10.1186/s12902-017-0159-5 (PMC5299777; doi:10.1186/s12902-017-0159-5)
Supplement: Additional file 1: Figure S1. — Flow chart of the Singapore Chinese Health Study. (DOCX 25 kb) [file 12902_2017_159_MOESM1_ESM.docx]

Additional file 1: Figure S1. Flow chart of the Singapore Chinese Health Study

Baseline (1993-1998)

Follow-up 1 (1999-2004)

Follow-up 2 (2006-2010)

Re-interviewed 52,325; 32,575 donated blood

Recruited 63,257 participants

Re-interviewed 39,528; 25,477 donated blood at follow-up 1

Case-control selection

Sample size: 571 cases and 571 controls

Cases: 1) donated blood;

2) reported no diabetes at blood donation;

3) reported to be diagnosed of diabetes at follow-up 2.

Controls: 1) donated blood;

2) reported no diabetes at follow-up 1 and follow-up 2;

3) HbA1c <6.0%.

Matching factors: age, gender, dialect group and date of blood collection
